# Diagnostics and Surgical Treatment of Deep Endometriosis—Real-World Data from a Large Endometriosis Center

**DOI:** 10.3390/jcm13226783

**Published:** 2024-11-11

**Authors:** Marcel Grube, Maren Castan, Alexander Drechsel-Grau, Teresa Praetorius, Karen Greif, Annette Staebler, Felix Neis, Katharina Rall, Bernhard Kraemer, Stefan Kommoss, Jürgen Andress

**Affiliations:** 1 Department of Gynecology and Obstetrics, University Hospital Tuebingen, Calwerstrasse 7, 72076 Tuebingen, Germany; 2 Department of Gynecology and Obstetrics, Diak Klinikum Schwaebisch Hall, Diakoniestrasse 10, 74523 Schwaebisch Hall, Germany; 3 Department of Urology, Cantonal Hospital St. Gallen, Rorschacher Strasse 95, 9000 St. Gallen, Switzerland; 4 Institute of Pathology and Neuropathology, University Hospital Tuebingen, Liebermeisterstraße 8, 72076 Tuebingen, Germany

**Keywords:** deep infiltrating endometriosis, endometriosis, diagnostics, surgery, pain, complications

## Abstract

**Background:** Deep endometriosis (DE) is a special form of endometriosis, one of the most common benign diseases in gynecology. In the specific case of DE, ectopic endometrium can be found not only in peritoneal but also in deeper tissue layers or even as parenchymal organ infiltration. Symptoms include dysmenorrhea, dyspareunia, dyschezia, and dysuria, as well as asymptomatic hydronephrosis or other organ dysfunctions. Due to a pathogenesis of the disease that has not been conclusively clarified to date, no causal therapy exists, which is why surgical resection of DE is still the gold standard for symptomatic cases. **Methods:** This article retrospectively describes the challenges in diagnosis and surgical treatment of DE at a German Level III Endometriosis Center, with a focus on diagnosis and surgical treatment, as well as the analysis of perioperative and postoperative complications. **Results:** The surgical treatment of DE is performed in most cases by minimally invasive laparoscopy (94.1%), whereas complex procedures such as ureterolysis, adhesiolysis, or preparation of the rectovaginal septum are considered standard procedures as well. The complexity of the procedures is further underlined by a high need for interdisciplinary operations (28%). Despite high complexity, severe postoperative complications occurred in only 3.1% of surgeries, with the complication rate being significantly higher whenever bowel surgery was necessary for DE resection. **Conclusions:** Our results emphasize the complexity and interdisciplinary nature of the disease. Therefore, treatment should preferably take place at an endometriosis center of the highest level with experienced, well-coordinated teams.

## 1. Introduction

Deep endometriosis (DE) is a special form of one of the most common benign diseases of premenopausal females with endometriotic lesions, which differs from other forms due to its infiltrative growth. DE is also associated with the co-existence of adenomyosis uteri, which is not evaluated in this study [1,2,3,4,5].

In many cases, patients report pronounced clinical symptoms such as pain or infertility [6]. Infiltration of the bowel or urinary tract can lead to stenosis and, in the worst case, to a secondary organ dysfunction like kidney failure [7,8].

Due to the heterogeneous clinical presentation, the diagnostics of DE are complex and can extend over several years [4,9,10]. Basic diagnostic tools such as clinical examination and transvaginal ultrasound [11,12] can be supplemented by other imaging modalities like MRI or CT in special cases, e.g., if the urinary tract is involved [11,13,14,15]. In addition to the possibility of histological confirmation of the diagnosis, nowadays laparoscopy as part of the diagnostic spectrum still offers the advantage of detecting even small or superficial endometriosis and allowing a precise statement to be made about its spread [14]. By using standardized staging classifications like #ENZIAN-Score, a standardized description of intraoperative findings is possible [16,17].

Currently, surgery is the only definitive treatment for DE. As the surgical spectrum for resection of DE includes complex procedures like ureterolysis, preparation of the rectovaginal space, or neurolysis of the hypogastric nerves, a high level of surgical expertise is crucial for successful surgery [18,19]. Further, bowel surgery such as disc resection (full-thickness resection of less than 50% of the circumference), shaving (superficial, layer-by-layer resection, preserving intestinal mucosa), or complete segment resection may also be required in many cases to reach complete resection, which underlines the need for interdisciplinary surgical teams [20]. Nevertheless, due to significantly reduced morbidity, surgery is performed laparoscopically or robotically assisted in most cases [21], with low complication rates of around 9% [22].

The aim of this study was to characterize the diagnosis and surgical treatment of DE at a large German Level III Endometriosis Center with a focus on diagnosis and surgical treatment, as well as the analysis of perioperative and postoperative complications.

## 2. Materials and Methods

We performed a retrospective chart review of patients surgically treated for DE to describe diagnostic as well as surgical procedures and to analyze peri- and postoperative complications, matching the following inclusion criteria: surgery for DE at the Tuebingen University Women’s Hospital between 1 January 2005 and 31 December 2015 and histopathological proof of at least one endometriotic lesion. DE had to be diagnosed according to at least one of the following criteria: histopathologically confirmed infiltration of (sub peritoneal) structures and/or visceral organs and/or indication of an ENZIAN score by the surgeon and/or clinical examination findings with evidence of DE and/or naming of procedures necessary for resection of DE procedures in the surgery report. For patients without histopathological evidence of infiltration, a random histopathological review was performed to prove DE.

A so-called index operation was defined for all patients. The index operation corresponds to the first treatment of DE during the observation period. All procedures performed within a period of six months prior to the index operation were considered diagnostic. Postoperative complications or follow-up interventions were assessed over a period of six months after the index surgery. All available electronic patient records including surgical and pathology reports were used for data collection.

Study data were collected and managed using REDCap electronic data capture tools hosted at Tuebingen University Women’s Hospital. REDCap (Research Electronic Data Capture) is a secure, web-based software platform designed to support data capture for research studies, providing (1) an intuitive interface for validated data capture; (2) audit trails for tracking data manipulation and export procedures; (3) automated export procedures for seamless data downloads to common statistical packages; and (4) procedures for data integration and interoperability with external sources [23,24].

Statistical analysis was performed using the following software: PRISM (GraphPad Software, version 9.0), REDCap (Vanderbilt, version 9.8.5), RStudio (RStudio PBC, version 1.4.1106). Descriptive statistics were applied, such as median, mean, and standard deviation. Comparison between groups was performed using a chi-square test for nominal variables and Student’s *t*-test and Mann–Whitney U-test for interval scaled variables, respectively, depending on the presence of a normal distribution. The significance level was assumed to be 5% throughout.

## 3. Results

### 3.1. Study Population

A total of 455 patients were available for the study. At the time of index surgery median age was 34 years (min. 16.69 years, max. 64.83 years) with a mean body mass index (BMI) of 23.3 kg/m^2^ (SD 3.93, min. 15.5, max. 40.1). 

A total of 226/455 (49.7%) patients were suspected of suffering from endometriosis at some point in their medical history prior to their first visit to Tuebingen University Women’s Hospital.

### 3.2. Symptoms and Indications for Surgical Therapy

At the time of index surgery, 438/455 (96.3%) patients suffered from symptoms typical for endometriosis, with pain being the main indication (68.4%) for surgery (Table 1). Other indications were, for example, asymptomatic hydronephrosis or hematochezia. In some cases, based on available patient records, the main indication remains unknown.

### 3.3. Preoperative Diagnostics

Transvaginal ultrasonography and nephrosonography were performed preoperatively in all patients. Abnormal findings were found in a total of 309/455 (67.9%) examinations. Ovarian cysts were found in 189/455 patients (41.5%), uterus myomatosus and suspicion of adenomyosis uteri were found in 50/455 patients (11.0%), respectively. Suspicion of DE in the rectovaginal septum was reported in 14/455 patients (3.1%).

A preoperative CT scan was performed in a total of 18/455 (4.0%) patients. In 16/18 (88.9%) cases, abnormal findings suspicious of DE were detected. Moreover, a total of 29/455 (6.4%) patients received an MRI. Thereby, 23/29 (79.3%) examinations showed abnormal findings compatible with DE.

A total of 156/455 (34.3%) patients underwent preoperative colonoscopy. This revealed a total of 29/156 (18.6%) abnormal findings suspicious of DE, like circular or partial stenosis or intraluminal lesions of endometriosis. Patients who suffered from gastrointestinal symptoms were statistically significantly more likely to have undergone a colonoscopy (*p* < 0.001) compared to patients without any gastrointestinal symptoms. However, there were no significant differences between the two groups with regard to the presence of pathological findings (*p* = 0.681).

Due to suspected involvement or infiltration of the urinary bladder, 36/455 (7.9%) patients underwent preoperative cystoscopy. Even though cystoscopy was performed statistically significantly more often in the presence of urological symptoms (*p* = 0.004), no significant difference with regard to pathological findings (*p* = 0.483) was detected.

A total of 264/455 (58.0%) patients underwent diagnostic laparoscopy within a period of six months prior to the index operation. The indication for invasive diagnostics was significantly more frequent if the diagnosis of endometriosis had not been confirmed in the past (*p* < 0.001). No intraoperative or postoperative complications occurred in the context of the diagnostic laparoscopy. Overall, a tissue sample was obtained for histological confirmation of endometriosis in 121/264 (45.8%) cases.

### 3.4. Gynecological Examination

Visual examination revealed suspicious findings of DE in a total of 78/455 (17.1%) patients. The main localization was the posterior vaginal wall. Further suspicious findings were found, e.g., at the portio (*n* = 4/455, 0.9%). In addition to the findings in the genital region, 3/455 (0.7%) cases showed endometriosis lesions in the umbilicus.

Palpation revealed signs of DE in a total of 321/455 (70.5%) patients. The most frequent localizations were the rectovaginal space in 44.8% of the cases as well as the sacrouterine ligaments in 22.0%. In addition to the two main localizations, there were isolated findings in the area of the abdominal wall (*n* = 5/455, 1.1%), the umbilicus (*n* = 3/455, 0.7%), and the caesarean scar (*n* = 3/455, 0.7%).

### 3.5. Surgical Access

Laparoscopy was chosen as the primary approach for a total of 428/455 (94.1%) surgeries. Only 21/455 (4.6%) patients underwent primary laparotomy, with a transverse approach being chosen in 90.5% of cases. With regard to the surgeries performed primarily by laparotomy, a clear correlation with the date of surgery can be seen as 61.9% of laparotomies were performed in 2005. 

After the primary laparoscopic approach, a change to laparotomy occurred in a total of 18/428 (4.2%). A total of 15/18 (83.3%) of the conversions occurred in the first half of the observation period from 2005 to 2010. The reason for a change of approach was the extension of the endometriotic findings in all cases, e.g., large bowel involvement or extended adhesions.

### 3.6. Intraoperative Findings

**Figure 1:**
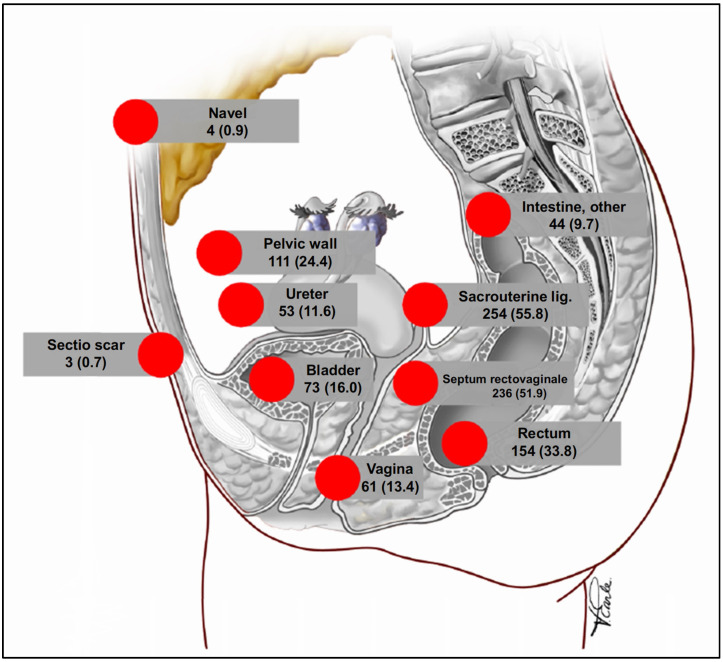
Schematic presentation of the localizations of deep endometriosis in the study population (*n* (%)), percentages related to the total population (*n* = 455), figure modified from Praetorius et al. [25].

The number of lesions of DE per patient was 1.98 (min. 1, max. 7) on average. Bowel endometriosis was found in a total of 174/455 (38.2%) patients, with endometriosis of the rectum (154/455, 33.8%) being the most common. Figure 1 shows the intraoperative dissemination of DE lesions within the study population. In addition to lesions of DE, a total of 324/455 (71.2%) patients had peritoneal or adnexal endometriosis.

The ENZIAN classification in its original form from 2005 [16] was used in a total of 137/455 (30.1%) cases to describe the intraoperative findings of DE. As another score to describe the intraoperative findings, the rASRM score was used in 171/455 (37.6%) cases. Other scores, such as the Endometriosis Fertility Score, were not used.

### 3.7. Surgical Procedures

**Figure 2:**
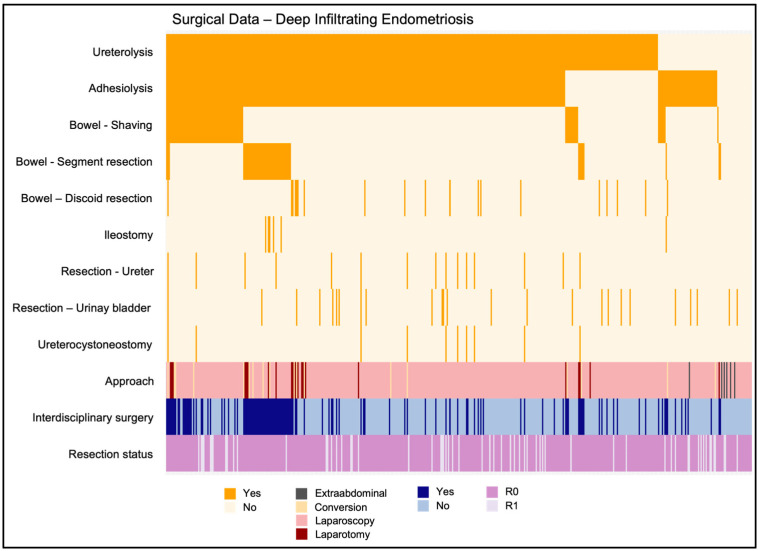
Surgical data of treating DE in index surgery, all patients (*n* = 455).

The most common procedure required in addition to endometriosis resection was unilateral or bilateral ureterolysis, which was performed in 382/455 cases (84.0%, Table 2). With similar occurrences, adhesiolysis was required in 356/455 (78.2%) cases. A detailed list of procedures is shown in Table 2 and Figure 2.

### 3.8. Interdisciplinary Surgeries

Interdisciplinary surgeries in cooperation with other departments were performed in 125/455 (27.5%) cases. The vast majority of these surgeries were performed with the support of a general surgeon in 81.6% (*n* = 102/125), and second most with urology in a further 12.0% (*n* = 15/125). Both of the above-mentioned departments were involved in 6.4% (*n* = 8/125) of the surgeries (Figure 2).

### 3.9. Complete Resection Rate

Complete resection of DE was achieved in 394/455 (86.6%) patients. In the remaining 61/455 (13.4%) cases, a complete resection was deliberately omitted (Figure 2). The main reason for incomplete resection was to avoid an extended intestinal intervention with the associated risk of the mostly anus praeter in 31/61 (50.8%) cases. In 8/61 (13.1%) patients, a complete resection of DE lesions was not performed because of missing clinical symptoms. In another 5/61 (8.2%) patients, the procedure was limited in favor of a quick realization of pregnancy. In order to avoid iatrogenic injury to the hypogastric plexus and associated micturition disorders, a complete resection was omitted in 5/61 (8.2%) patients. 

The majority of residual DE lesions were found on the rectum or the rectovaginal septum with 28/61 (45.9%) cases, followed by the uterosacral ligaments (*n* = 12/61, 19.7%), other parts of large or small intestine (*n* = 8/61, 13.1%), and the area of the urinary bladder (*n* = 7/61, 11.5%).

### 3.10. Duration of Surgery and Inpatient Stay

The mean duration of surgery was 156.12 min (min. 21 min, max. 752 min). Several factors had a significant influence on duration, such as interdisciplinary surgery. Whereas a surgery performed exclusively by gynecologists took an average of 110.8 min (min. 21 min, max. 300 min), the duration of surgery increased to 265.2 min (min. 80 min, max. 664 min) in the case of cooperation with another department, and even to 425.3 min (min. 240 min, max. 733 min, *p* < 0.001) in the case of cooperation with two other departments.

Except for 11/455 (2.4%) patients, DE resection was performed during an inpatient hospital stay. The mean length of stay was 5.37 days (min. 1, max. 43,). Again, there was a statistically significant correlation with the involvement of other departments. While the inpatient stay of patients after purely gynecological procedures was 3.87 days, it increased to 8.9 days after intraoperative cooperation with another department or to 14.3 days (*p* < 0.001) with more than one other department.

### 3.11. Intraoperative Complications

Intraoperative complications occurred in 2/455 (0.4%) patients. Both patients had an iatrogenic injury of the colon, which was treated by suturing after the involvement of a general surgeon. One of the patients had an intraoperative drop of hemoglobin to 5.7 g/dL due to severe intraoperative bleeding with the need for a blood transfusion. The postoperative process was without complications in both patients.

### 3.12. Postoperative Complications

Overall, 50/455 (11.0%) patients experienced postoperative complications within the first six months after index surgery. According to the classification per Clavien and Dindo [26], there were second-degree complications in particular (*n* = 19/50, 38.0%). Readmission was necessary in 14/50 (28.0%) cases.

Postoperative infection was the most common complication, requiring treatment in 22/50 (44.0%) cases. The urinary tract was particularly frequently affected (*n* = 14/50, 28.0%). Postoperative fever occurred in 6/50 (12.0%) cases. Besides the 1/50 (2.0%) infected surgical wound with a resulting wound healing disorder, there were 2/50 (4.0%) additional pelvic abscesses that needed to be relieved surgically. All patients with postoperative infection received antibiotic therapy. In 4/50 (8.0%) patients, the development of a postoperative hematoma resulting in a decrease in hemoglobin was a reason for readmission. Surgical revision was necessary in all of these cases.

A total of 19/50 (38.0%) patients suffered postoperatively from functional disorders in the urinary tract. In 7/50 (14.0%) cases, bladder dysfunction occurred, resulting in 1/50 (2.0%) patients having to catheterize themselves. While 3/50 (6.0%) patients had to undergo surgical revision due to postoperative urinary retention, it was necessary in 2/50 (4.0%) cases due to a lesion of the urinary bladder.

There was a significantly higher complication rate after bowel surgery (*n* = 27/174, 15.5% vs. *n* = 23/281, 8.2% without bowel surgery, *p* = 0.020). In comparison to the DE resection by shaving or discoid resection, there was an accumulation of postoperative complications after bowel segment resections (*n* = 13/97, 13.4% vs. *n* = 15/48, 31.3%, *p* = 0.014). Surgical revision of anastomotic insufficiency was required in 3/48 (6.3%) cases; in two patients, a protective ileostomy was needed.

Performing a colpotomy was also statistically significantly associated with an increased rate of complications (*n* = 25/111, 21.6% vs. *n* = 25/313, 7.4% without colpotomy, *p* < 0.001). Suture dehiscence in the vagina or recto-/vesicovaginal fistulas occurred exclusively after colpotomy in only 1/111 (0.9%) and 3/111 (2.7%) patients, respectively.

### 3.13. Histopathological Findings

A total of 1615 specimens were sent for histopathological evaluation, with an average of 3.55 specimens per patient (min. 1, max. 13). A total of 76.5% (*n* = 1235/1615) of all specimens contained endometriotic tissue, whereas endometriosis could be histologically confirmed in at least one specimen of each patient.

In 184/455 (40.4%) patients, the histopathological findings showed infiltration of the subperitoneal structures or visceral organs. Structures infiltrated by DE were the bowel (*n* = 85/184, 46.2%), the vagina (*n* = 41/184, 22.3%), the pelvic muscles and connective tissue (*n* = 40/184, 21.7%), as well as the urinary bladder (*n* = 36/184, 19.6%). Extra abdominal manifestations were evident in the umbilicus (*n* = 4/184, 1.1%).

## 4. Discussion

Endometriosis in general not only poses special challenges for patients, their personal environment, and the physicians treating them, but also represents a substantial financial problem for the healthcare system, resulting in a considerable financial burden [27]. At the same time, many questions remain unsolved, especially regarding the specific form of DE. The aim of this study was to describe the challenges and requirements for diagnostics and surgical treatment of DE in a large German endometriosis center. It was the aim to provide a contribution to the understanding of DE and to gain new insights that could help physicians to improve the counseling and treatment of patients.

Although currently imaging modalities are increasingly advocated and ubiquitously available in the diagnosis of DE [28], careful gynecological examination and qualified ultrasonography by the clinically and surgically experienced gynecologist is still an essential part of the diagnostic work-up in cases suspected of endometriosis [12]. This is followed by laparoscopy for histological confirmation of disease and treatment of symptomatic DE [29]. The use of imaging diagnostics such as MRI and CT is rather low, with significantly less than 10%, in the present study, which is collectively explained by the selected time period (2005–2015). The first reliable studies on the use, benefit, and correlation with intraoperative findings of various diagnostic devices were published towards the end of the observation period. Due to further technical development and increasing experience in the diagnosis of endometriosis findings, especially MRI is now a valuable diagnostic method for suspected DE whenever expert transvaginal ultrasound raises specific questions. Although MRI, sonography, and clinical examination can provide important clues about the extent of the disease, key aspects of direct inspection of the peritoneal cavity are left out. Diagnostic laparoscopy offers the surgeon and his interdisciplinary team the opportunity to palpate indurations, assess adhesions between anatomical structures, and evaluate the exact extent of endometriosis, especially for example bowel or ureteral involvement [30]. Then, diagnostic laparoscopy can be followed up by a well-considered and individualized laparoscopic intervention for resection of DE. Nowadays, thanks to the already described improvement in imaging technologies, diagnostic laparoscopy would play a less important role and would not have been necessary in most cases. In many cases, the latest ultrasound techniques in the hands of an endometriosis expert can already provide all the information needed for DE surgery planning. 

Furthermore, recent studies show promising results in using microRNA as a biomarker for the diagnosis of endometriosis in the future [31]. The development of a blood test for endometriosis that can be performed easily in an outpatient setting could be the next big step to simplify diagnostics and to help reduce the time for patients to receive the diagnosis and appropriate treatment of endometriosis.

As the rate of complications rises with the increasing extent of endometriosis surgery, the risks and possible benefits of the surgical treatment must be discussed in detail with the patient preoperatively, and together, an individualized treatment strategy must be developed. The more information on the extent of the disease the physician has, the better he can advise the patient regarding further treatment. Even if resection of DE lesions can reduce pain symptoms and improve fertility in a majority of patients, an individualized approach based on the patient’s symptoms, wishes, and possible risks is always necessary. In some cases, it may be appropriate to deliberately not resect all DE lesions in order to minimize the risk of postoperative complications, particularly in the absence of clinical symptoms.

As our study also shows, DE resection can be a very complex surgery. Particularly against the background of benign disease, nerve-sparing surgery, dissection of the ureter, or bowel procedures are among the most common procedures that an endometriosis surgeon should be able to perform; however, especially in the case of bowel surgery, it is still not clear which form of resection (disc resection vs. shaving vs. segment resection) is the best in terms of symptom control, recurrence, and complication rate. Despite their complexity, DE surgery can be performed laparoscopically, with serious complications (Clavien Dindo ≥ 2) occurring in only 3% of our patients, which is consistent with the data from the literature [32].

In the near future, robotic-assisted surgery may be able to further improve surgical outcomes due to its higher precision, particularly for complex surgical procedures in tight pelvic spaces.

This study provides important arguments for the treatment of DE at an experienced endometriosis center, which has been recommended both by the Endometriosis Research Foundation and by the German guidelines. This allows for surgical treatment of DE with the best possible expertise, and therefore, low complication rates. Furthermore, in line with current literature, DE cannot only be considered a gynecological disease alone [33]. Only an interdisciplinary therapeutic approach involving all specialist disciplines at a very early point of diagnosis can provide the best treatment for the patient. Therefore, there is a big need for the establishment of interdisciplinary endometriosis case conferences, which is already standard procedure in oncology. Well-structured and standardized staging examinations as well as interdisciplinary preoperative case demonstrations could improve the treatment of DE patients.

In the future, another focus must be placed on the consistent training of young surgeons in demanding laparoscopies. As our study shows, surgical treatment of DE requires exceptionally difficult, minimally invasive skills, which require a lot of supervised surgical training. Only with a well-educated and experienced team can surgical treatment be guaranteed at the highest level for as many patients as possible. Here too, robotic-assisted surgery can contribute to optimizing DE treatment thanks to a shorter learning curve compared to standard laparoscopy [34].

Although the results of our study support and confirm other publications, one of the main limitations results from its retrospective design. As there is no follow-up data available, this study is not able to tell anything about the long-term results of DE surgery regarding pain improvement and fertility. Furthermore, non-systematic follow-up may limit the significance of our results regarding postoperative complications. As the awareness of and knowledge about the disease has continuously developed over the observation period, there was no systematic clinical documentation of endometriosis parameters in the patient records. Therefore, defining the study population was especially difficult. In the absence of a uniform definition of DE that has remained constant over the years, it was necessary to establish a definition and apply it accordingly. Furthermore, all data shown is from a single-center study, which may limit the generalizability of our findings.

Overall, our study underlines the complexity of the diagnosis and treatment of DE. The results show that surgical treatment should be carried out at a center with the appropriate experience and expertise. It is also clear that adequate treatment can only be provided on an interdisciplinary basis. In an appropriate setting, the surgical treatment of DE can be carried out with low complication rates despite its complexity.

Even though research on endometriosis has increased significantly in recent years, there is still an urgent need to intensify research on DE and endometriosis in general, not only due to an increasing number of patients suffering from this disease.

## Figures and Tables

**Table 1:** Patients’ characteristics, clinical symptoms, and indication for DE surgery (all percentages related to the complete study cohort *n* = 455).

Variable	
Patients’ characteristics	
Age at index surgery, years ^a^	34 [16.7–64.8]
Body mass index at index surgery, kg/m^2 b^	23.3 [3.93, 15.5–40.1]
Symptomatic patients	438 (96.3%)
Patients’ symptoms	
Dysmenorrhea	343 (75.4%)
Dyspareunia	200 (44.0%)
Lower abdominal pain	185 (40.7%)
Dyschezia	149 (32.7%)
Hematochezia	23 (5.1%)
Dysuria	58 (12.7%)
Hematuria	3 (0.7%)
Urinary retention	
Asymptomatic	15 (3.3%)
Symptomatic	5 (1.1%)
Infertility	164 (36.0%)
Main Indication for surgery	
Pain	311 (68.4%)
Infertility	88 (19.3%)
Risk of organ failure (hydronephrosis, intestinal stenosis)	22 (4.9%)
Other	17 (3.7%)
Unknown	17 (3.7%)

^a^ Data are characterized as median [range]; ^b^ Data are characterized as mean [SD, range].

**Table 2:** Surgical procedures for resection of DE (all percentages related to the complete study cohort *n* = 455).

Variable	
Procedures—gynecologic	
Resection DE	455 (100.0%)
Ureterolysis	382 (84.0%)
Adhesiolysis	356 (78.2%)
Colpotomy	111 (24.4%)
Hysterectomy	51 (11.2%)
Adnexectomy	37 (8.1%)
Neurolysis hypogastric nerve	18 (4.0)
Procedures—interdisciplinary	
Bowel—shaving	77 (16.9%)
Bowel—discoid resection	20 (4.4%)
Bowel—Segment resection/anastomosis	48 (10.5%)
Ileostomy	6 (1.3%)
Appendectomy	10 (2.2%)
(Partial) resection—ureter	15 (3.3%)
(Partial) resection—urinary bladder	25 (5.5%)
Ureterocystoneostomy	10 (2.2%)
Psoas hitch	2 (0.4%)

## Data Availability

Data is available upon request from the corresponding author.
